# Cor Triatriatum Sinister Presenting as Cardioembolic Stroke in a Young Woman

**DOI:** 10.3390/diagnostics13010097

**Published:** 2022-12-29

**Authors:** Timea Magdolna Szabo, Erhard Heidenhoffer, Ádám Kirchmaier, Benedek Pelok, Attila Frigy

**Affiliations:** 1 Department of Biochemistry and Environmental Chemistry, George Emil Palade University of Medicine, Pharmacy, Science, and Technology of Targu Mures, 540142 Targu Mures, Romania; 2 Department of Cardiology, Clinical County Hospital Mures, 540103 Targu Mures, Romania; 3 Department of Neurology, Municipal Hospital Odorheiu Secuiesc, 535600 Odorheiu Secuiesc, Romania; 4 Department of Internal Medicine IV, George Emil Palade University of Medicine, Pharmacy, Science, and Technology of Targu Mures, 540103 Targu Mures, Romania

**Keywords:** cor triatriatum sinister, cardioembolic stroke, congenital heart disease

## Abstract

Cor triatriatum sinister is a rare congenital heart disease characterized by an additional fibromuscular membrane in the left atrium. Cardioembolic stroke is a rare complication of cor triatriatum sinister, especially among women. We hereby describe the case of an 18-year-old female patient, without a past medical history, presenting with cardioembolic stroke in the territory of the right posterior cerebral artery. During extensive diagnostic work-up, nonrestrictive cor triatriatum sinister and patent foramen ovale were diagnosed using transthoracic and transesophageal echocardiography. In clinical practice, it is important to identify congenital cardiac defects as potential substrates for cardioembolism in young patients. In our case, cor triatriatum sinister presenting as ischemic stroke was diagnosed, which is an uncommon finding, especially in young females. Determining the optimal management strategy for patients with cor triatriatum sinister complicated by cardioembolic stroke requires a multidisciplinary approach.

Cor triatriatum sinister (CTS) is a rare congenital heart anomaly defined by an accessory fibromuscular membrane dividing the left atrium (LA) into proximal and distal chambers. The pulmonary veins usually drain into the upper, accessory LA, whereas the lower, real LA communicates with the left atrial appendage (LAA) and mitral valve. The two LA compartments connect via openings of variable size and number. CTS accounts for up to 0.4% of all congenital heart disease (CHD) [1]. It frequently accompanies other CHD, such as ostium secundum atrial septal defect (ASD), patent foramen ovale (PFO), anomalous pulmonary venous return, left superior vena cava, congenital mitral valve regurgitation, pulmonary artery stenosis, double outlet right ventricle, tetralogy of Fallot, and it has been previously described in this regard [2]. A higher incidence among males has been reported [3]. Moreover, the association between orofacial clefting and CTS has been frequently encountered among consanguineous Amish and Saudi Arabian communities, suggesting an autosomal recessive inheritance [4]. 

The most widely accepted hypothesis for the formation of CTS is the theory of malincorporation, which ascribes the accessory membrane to the abnormally integrated common pulmonary vein [2]. Mutations in HYAL2 genes leading to hyaluronidase 2 deficiency might be a potential contributor to CTS [4,5]. Hyaluronidase 2 breaks down high molecular weight hyaluronan (HA) into smaller fragments. The resulting low molecular weight HA plays an important role in the regulation of epithelial-mesenchymal transition (EMT) and normal cardiac chamber formation [6]. In hyaluronidase 2 deficiency, excess HA may induce overt EMT and lead to redundant fibromuscular tissue [4,5].

CTS may present as an incidental finding during cardiac imaging examinations or may be associated with various manifestations. The number and size of membrane fenestrations determine the degree of obstruction between the left chambers of the heart and, along with the accompanying congenital cardiac anomalies, dictate earliness and severity of symptoms. 

We hereby describe the case of a patient with previously asymptomatic CTS who presented with ischemic stroke.

An 18-year-old woman was referred to the cardiology department in search of potential cardiac sources of embolism after suffering an ischemic stroke in the territory of the right posterior cerebral artery (Figure 1). 

The patient denied having a family history of cardiovascular disease and symptoms of cardiac origin. She had an unremarkable medical history, and her physical examination was normal. Resting 12-lead electrocardiogram showed sinus rhythm with normal heart rate and no morphological anomalies. As part of the diagnostic work-up, transthoracic echocardiography was also carried out, revealing normal cardiac chamber size, left and right ventricular function, and no valvular heart disease. An echodense structure was identified bisecting the LA horizontally into postero-superior and antero-inferior parts. No significant pressure gradient was detected using continuous Doppler across the flexible membrane in the four-chamber view (Figure 2).

In order to better visualize the identified structure within the LA, a transesophageal echocardiography was performed. The membrane stretched out from the interatrial septum right beneath the foramen ovale to the lateral atrial wall above the LAA (Figure 3). 

Multiple membrane fenestrations ensured the connection between the proximal and distal LA chambers. Color Doppler interrogation of the interatrial septum revealed PFO with minimal spontaneous left-to-right shunt (Figure 4).

No evidence of right-to-left shunt with agitated saline injection was identified during the Valsalva maneuver. No echodense structures indicative of thrombus or spontaneous contrast were visualized within the LA and LAA. Repeated 24-h Holter monitoring did not detect any atrial or ventricular arrhythmias. Given the echocardiographic findings, cardioembolic stroke was highly suspected. Our patient started oral anticoagulation (OAC) with 150 mg of dabigatran, b.i.d., and was referred for surgery. She opted for conservative management, continuing medical treatment without stroke recurrence during follow-up.

CTS is commonly diagnosed during infancy and early childhood, presenting as left-sided heart failure, secondary pulmonary hypertension, poor growth, or arrhythmia [7]. Only a few cases of stroke have been previously reported in CTS patients [8,9,10,11,12,13,14,15,16,17,18], and, to the best of our knowledge, only one of a young female (besides our patient) [9]. In that case, as opposed to ours, the patient presented with recurrent ischemic stroke despite antithrombotic therapy with aspirin and later on with 5 mg of apixaban, b.i.d. [9]. This highlights the importance of proper patient selection for anticoagulation. Surgical excision of the fibromuscular diaphragm is the standard of care in most cases of CTS, especially when associated with complex CHD [7]. Several surgical centers reported excellent long-term survival rates after membrane resection with minimal risk of postoperative reintervention [19,20]. When surgical repair is not feasible or preferred, the percutaneous approach for LA decompression provides an acceptable alternative. Blais et al. managed to improve left ventricular inflow hemodynamics using a transseptal catheterization procedure by puncturing the obstructive membrane utilizing radiofrequency energy prior to performing balloon dilation at the site of the newly formed connection [21]. This option may help pregnant patients with previously undiagnosed or conservatively managed CTS presenting as acute heart failure triggered by physiological changes in pregnancy [21]. Our patient continued OAC, although surgery would have been the optimal therapeutic management, given the low operative risk, the possibility of future pregnancy-associated complications, and anticoagulation-related bleeding events. CTS may predispose patients to supraventricular arrhythmias due to abnormal LA anatomy and remodeling [22]. Patients with unrestrictive CTS and drug-resistant left atrial tachyarrhythmias may benefit from single- and multi-chamber catheter ablation techniques [22]. Provided that our patient had no history of palpitations and did not present atrial tachyarrhythmias during repeated 24-h Holter monitoring, in situ LA thrombus formation or paradoxical embolism via PFO accounted for possible causes of ischemic stroke. If opting for surgery, the closure of PFO/ASD would also have been carried out. The percutaneous closure of PFO/ASD is another option in patients with associated nonrestrictive CTS and optimal atrial anatomy [23]. 

Cor triatriatum is a rare but increasingly recognized condition. Although the routine and systemic use of echocardiography as an imaging modality helps establish the correct diagnosis, further therapeutic management imposes several questions regarding treatment decisions concerning open-heart surgery versus percutaneous approach, thromboembolic prophylaxis, and follow-up, especially when embolic complications occur.

## Figures and Tables

**Figure 1:**
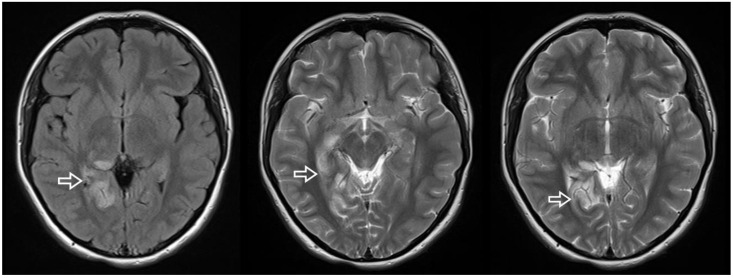
Brain MRI axial sections showing the ischemic lesions in the territory of the right posterior cerebral artery (arrow).

**Figure 2:**
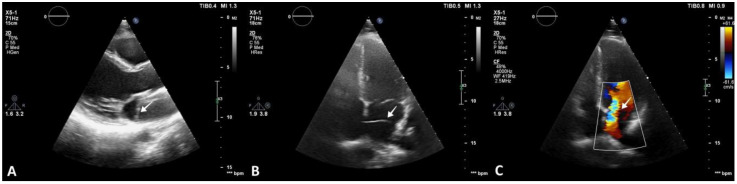
Transthoracic echocardiography revealing CTS. (**A**) Parasternal long-axis 2D view showing an echodense, membranous structure within the LA (arrow). (**B**) Apical four-chamber 2D view showing the membrane bisecting the LA (arrow). (**C**) Apical four-chamber 2D view and color Doppler window placed over the LA, highlighting turbulent flow across the membrane (arrow).

**Figure 3:**
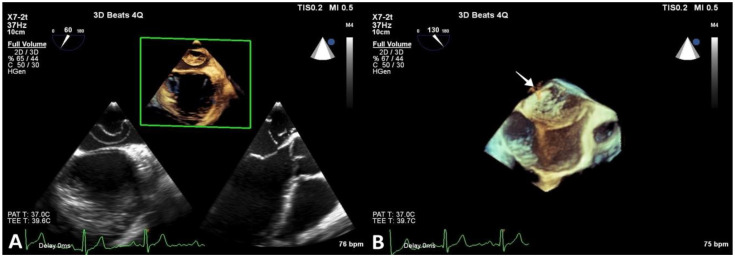
Transesophageal echocardiography revealing CTS. (**A**) and (**B**) show 2D and 3D reconstructions visualizing the membrane and its fenestrations (arrow).

**Figure 4:**
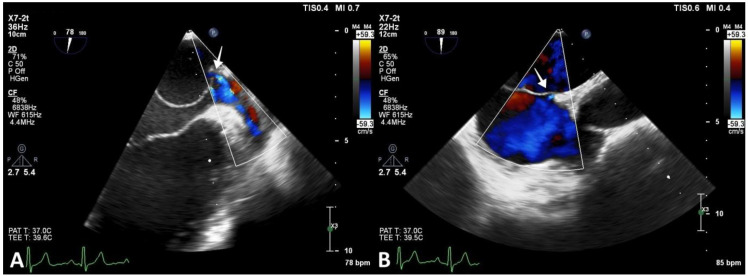
Transesophageal echocardiography revealing CTS. (**A**) Image showing openings in the CTS membrane (arrow). (**B**) PFO with minimal spontaneous left-to-right shunt (arrow).

## Data Availability

Not applicable.
